# To Junior Dentists

**Published:** 1885-12

**Authors:** 


### TO JUNIOR DENTISTS. NO III.

### DEVITALIZING PULPS.

*My Dear Doctor:*

There is scarce one of us who is able to free himself from the dominion of other minds. Reason, experience, observation, all go for nought in the presence of the dictum of one whom we have long looked upon as an authority. We cling to the most absurd superstitions, we accept as infallible truths the wildest speculations, we follow in practice the most preposterous hypotheses, if only they are generally acknowledged as quite orthodox. If a man once be accepted as a leader and then be prodigal enough of positive declarations, if he continue, in season and out of season, to loudly assert his own infallibility and proclaim the assinine ignorance of every other man, he will find enough of the unthinking to blindly follow him, though he be at all times incoherent, inconsistent, and as changeable as a weather-cock. If he shall have written a book, he will need do no more than to dogmatically assert that he alone is the true teacher, and there will be fools enough to swear by him, though no two chapters are compatible. The amount of it is, few men care to think for themselves. Consistent reasoning requires considerable effort, and it is so much easier to obtain theories at second-hand that most men shirk the labor, even if they are competent to perform it. This is severely true in dental practice, and hence the most of us are mere copyists, obtaining our ideas and methods from those who are more dogmatic and positive than we are. So true is it, that I doubt if there are many who will take the trouble to thoroughly comprehend this prologue.

I give you credit for something better, for your letter of inquiry concerning the destruction of tooth-pulps seems to demand law and reason, and not the mere dictum of any man, or set of men. It has been loudly proclaimed that any dentist who wilfully destroys a tooth-pulp, or even the last moribund vestige of one, commits a henious crime. If this be so I am a villain of the blackest dye, and steeped in iniquity to the lips, for I have destroyed many. Probably I am about the only sinner in the profession, for though we hear very much in societies and the journals about the treatment of root-canals, no one admits that he was responsible for the death. Dentists have heard pulp devitalization so roundly denounced, and in such scathing terms, that they dare not practice it. But I am ready for immolation, and hereby freely admit that I destroy pulps, and use arsenical paste for that purpose, too.

I would not have you understand that I indiscriminately destroy every pulp that I meet in practice. Far from it. There are few drugs in our pharmacopœia that have been more abused than arsenious acid, but in my opinion it has its legitimate uses. And now comes the question, when is its use justifiable, and when to be condemned? Ah, my dear doctor, that is a matter which cannot be explained in a paragraph. As a young dentist, you are, doubtless, like most of the others, searching for specific, definite rules. You wish a perfect law, that shall teach you, in every case that presents, precisely what your duty is. You desire an unerring guide that, irrespective of any complications, will dictate what is good practice. I know the feeling, for I have been there myself. For years I studied recipes and specifics, until it finally dawned upon my obtuse understanding that this was, in principle, quackery of the worst kind. When you are older, and have a more perfect acquaintance with physiological laws, you will comprehend that there are many factors which enter into the sum of even the simplest disease. No man has mastered them all, and probably no man ever will. Should any one do so, that will be the end of progress so far as he is concerned. He will have nothing more to learn, and will be quite ready for translation.

You will find many panaceas, and specifics, and universal laws and remedies advertised in the annals of charlatanism, but you will never try any of them but once. Perpetual motion, the philosopher's stone, and the perfect catholicon will all be discovered together.

When I say that certain pulps should be destroyed and certain others treated for preservation, I cannot definitely draw a clear line of demarcation that shall enable you to determine them at a glance, although I have my own ideas concerning them. I can usually de-determine a case for myself, after due inquiry. How do I do it? I examine it in the light of more than twenty years' experience, my boy. Have I lived and practiced all these years for nothing? It has been the study of my waking hours and the dreams of my bed at night, to determine these vexed questions. Have I accomplished this? No! a thousand times, no! But I have made some progress. Years ago, when at a Dental Society Meeting I heard oxy-chloride of zinc vaunted as an infallible remedy for exposed pulps, I went home and, in all honesty and anxious earnestness, faithfully experimented. For some time I was in ecstasies, for I thought the dental millennium had come, and the Savior of decayed teeth reigned on earth. But when the antiseptic qualities of the filling were exhausted, and the dead pulp manifested itself in a discolored tooth, an absorbed alveolus, and an abscessed periosteum, in my heart I cursed the mistaken enthusiasts who had led me astray. It is not a very pleasant reflection to know that perhaps others are anathematizing me for assertions more zealous than wise. But, like most people, I am constantly straying off in the lead of false prophets, and presume I shall be to the end. My office is full of patent gimcracks for the accomplishment of impossible things. If at some time a fluent talker shall come along with a set of cog-wheels for insertion in the molar teeth, to aid in the more perfect mastication of food, I dare say that I shall be a purchaser, despite good resolutions.

But you desire to know something about my practice in the devitalization of pulps. (Please never use the term "nerve" in this connection. It is a horrid solecism.) When an exposed pulp presents itself, there are many questions that I must ask myself before I can come to a decision. I must take into consideration all the circumstances. If the exposure be recent and traumatic, the result perhaps of careless excavating, that is one indication for treatment with a view to its preservation. If the patient be young and vigorous, that is another. If the mouth and oral tissues be in a general state of health, and the alveolus be not materially absorbed, that is another. If the cavity of decay be not very extensive, that is yet another. Every indication that the patient is in a good condition for resistance to disease, is an argument for the preservation of the pulp.

But when a case is presented in which the patient is pale, anæmic, in a low state of vitality, perhaps suffering from some chronic disease, that is an indication for devitalization. If of advanced age, the processes of repair are inactive, as they are in diseased conditions, and that is another reason. If there be a markedly septic condition of the mouth, it will be difficult to restore the pulp to a healthy state. If, for purposes of contour, it be necessary to insert a very large metallic filling, the continued thermal changes will be inimical to the continued life of the pulp, even though it be once successfully capped. If there be extensive or material absorption about the roots of a tooth with an exposed pulp, it will be very difficult to preserve it. If the tooth stand alone, it will be liable to greater shocks, and hence increased danger to an affected pulp, than if it is protected by adjoining teeth. Finally, the condition of the pulp itself will have much to do with the promise of a successful capping. If there be much inflammation and considerable engorgement, it is, in most cases, hopeless to attempt its salvation. You may not be able to judge concerning this by appearances, for it is seldom that a pulp is so largely exposed as to give a view that shall enable you to fully determine. But the patient will tell you. If there shall have been considerable pain, and especially if it shall have been of long standing, unless the circumstances are unusually favorable, there is little hope from capping. If there be a kind of soreness to the tooth, not the result of periostitis, or if there be any great degree of sensitiveness to thermal changes, these are unfavorable indications. It should be noted that sensitiveness to cold is one of the earlier symptoms of an inflamed and irritated pulp. When this has changed to intolerance of heat, while cold gives relief, the pulp is in its last stages, and radical extirpation is loudly demanded. You will occasionally find pulps that have long been extensively exposed without pain. They bleed at the slightest touch, but are not sensitive. These would look like favorable cases for capping, and sometimes they are. But make very sure that there is no fungoid, vascular growth, which you mistake for a living pulp.

You will thus see that there are many things to be taken into the account in determining what you will do with an exposed pulp. Look the ground over very carefully. Your mind once made up, proceed firmly, though cautiously. If the pulp must be destroyed, put on the rubber dam and clean the debris out of the cavity as perfectly as possible. Take the least perceptible portion of arsenious acid preparation, and apply it directly to the pulp, and then seal up the cavity effectually with wax, gutta-percha, or anything that will surely retain your medicament. Remember that if it ooze out and come in contact with the soft tissues, it will be likely to cause a bad ulcer. If it reach hard tissue, it may result in necrosis. Should you unfortunately find traces of it outside the cavity of decay, use dialyzed iron to neutralize it.

I usually leave the paste in the tooth about forty-eight hours, but you will frequently find that six hours have completed the work, if it be well applied. Then remove it, clean out and open up the pulp cavity, and insert for twenty-four hours a pledget of cotton wet in carbolic acid and dipped in tannic acid. This will contract and toughen the pulp, so that its removal intact will be more easy.

I have removed pulps painlessly by the use of a saturated solution of muriate of cocaine, but this has not crystallized into a settled practice with me as yet.

